# Editorial: Untangling post-treatment follow up of brain tumors: the role of neuroimaging

**DOI:** 10.3389/fradi.2023.1204517

**Published:** 2023-06-12

**Authors:** Nicoletta Anzalone, Letterio S. Politi, Massimo Caulo

**Affiliations:** ^1^ Vita e Salute University, San Raffaele Hospital (IRCCS), Milan, Italy; ^2^ Neuroradiology Department, San Raffaele Hospital and Vita e Salute University, Milan, Italy; ^3^ Humanitas University, Pieve Emanuele, Milan, Italy; ^4^ IRCCS Humanitas Research Hospital, Rozzano, Milan, Italy; ^5^ University of Studies G. d'Annunzio Chieti and Pescara, Chieti, Italy; ^6^ Radiology Department, D'Annunzio University, Chieti, Italy

**Keywords:** follow up of brain tumors, treatment effect, radionecrosis, perfusion, MR

**Editorial on the Research Topic** Untangling post-treatment follow up of brain tumors: the role of neuroimaging

One of the most challenging issues that a neuroradiologist must face in brain tumor surveillance is to differentiate disease progression from treatment effects. This issue is well-known, and magnetic resonance imaging (MRI) with conventional acquisitions using gadolinium has reached its limits (1). A correlation between MRI timing with treatment phase has been shown to help with differential diagnosis (2), but the presence of late-onset cases render conventional imaging inconclusive. After introduction of the STUPP treatment protocol (3) as well as new immunotherapies for high-grade gliomas, treatment-related effects due to combined chemoradiotherapy have become increasingly present. The notorious “pseudoprogression,” which involves an excessive inflammatory response that may mimic disease progression, is the hallmark of these treatment-related changes (4, 5). As for the effects of the monotherapy with irradiation, a correlation with the therapy timing may not provide diagnostic clues; pseudoprogression can also happen outside the more frequently described period (3–6 months) after treatment commencement. The limitations of conventional gadolinium-enhanced MRI have been very well described, and several recent studies have highlighted the merits of advanced MR techniques for the primary staging of brain tumors and their effective surveillance (6).

This special issue includes two reviews that describe in depth the role of MR advanced techniques such as perfusion, diffusion, and spectroscopy to differentiate disease progression from treatment effects in brain tumors with an emphasis on gliomas. Li and Iv considered the different potentials of each MR acquisition in their didactic review using representative cases. Malik et al. focused on the technical aspects of perfusion, especially those of dynamic susceptibility contrast-enhanced MRI, stressing the importance of relative cerebral blood volume thresholds to distinguish tumor progression from post-treatment radiation effects. In addition, the potential role of artificial intelligence along with machine learning and deep learning as diagnostic aids has been discussed, and the advantages of positron emission tomography (PET), particularly the radiolabeled amino acid PET-tracers, have been reported (7). Li and Iv describe in detail their state-of-the-art use and role in differentiating disease progression from treatment effects. Moreover, the discussion dives beyond the STUPP protocol into the utility of advanced imaging techniques in patients receiving antineoangiogenic treatments such as bevacizumab, which is a popular second-line treatment for glioblastomas (8). Their effect on imaging findings through modification of the blood-brain barrier leads to shortcomings in the gadolinium-enhanced sequences and underpins the value of unenhanced acquisitions such as FLAIR and DWI (9). Gaudino et al. reported on MRI findings after regorafenib treatment, another antineoangiogenic molecule that has not previously been described in detail. Interestingly, two different patterns in MRI findings emerged during this treatment regimen, each related to a different prognosis. Furthermore, a case of a germinal thalamic tumor that showed spontaneous but transient regression after treatment with steroids is presented. This is a remarkably unusual case, indicating that response to treatment may also depend on a complex immune reaction cascade.

In conclusion, the contents of this special issue will provide the reader with useful and up-to-date information on how we can address the diagnostic challenges in the post-treatment imaging of brain tumors, a notoriously difficult task that can be made easier using advanced imaging techniques.

## References

[1] Kumar AJ Leeds NE Fuller GN Van Tassel P Maor MH Sawaya RE Malignant gliomas: MR imaging spectrum of radiation therapy and chemotherapy induced necrosis of the brain after treatment. Radiology. (2000) 217:377–84. 10.1148/radiology.217.2.r00nv36377 11058631

[2] Pruzincova L Steno J Srbecky M Kalina P Rychly B Boljesikova E MR imaging of late radiation therapy and chemotherapy induced injury: a pictorial essay. Eur Radiol. (2009) 19:2716–27. 10.1007/s00330-009-1449-8 19471942

[3] Stupp R Mason WP van den Bent MJ Weller M Fisher B Taphooorn MJB Radiotherapy plus concomitant and adjuvant temozolamide for glioblastoma. New Engl J Med. (2005) 352:987–96. 10.1056/NEJMoa043330 15758009

[4] Brandsma D Stalpers L Taal W Sminia P van den Bent MJ. Clinical features, mechanism, and management of pseudoprogression in malignant gliomas. Lancet Oncol. (2008) 9(5):453–61. 10.1016/S1470-2045(08)70125-6 18452856

[5] Thust SC van den Bent MJ Smits M. Pseudoprogression of brain tumors. J Magn Reson Imaging. (2018) 48(3):571–89. 10.1002/jmri.26171 29734497 PMC6175399

[6] Strauss SB Meng A Ebani EJ Chiang GC. Imaging glioblastoma posttreatment progression, pseudoprogression, pseudoresponse, radiation necrosis. Radiol Clin N Am. (2019) 57(6):1199–216. 10.1016/j.rcl.2019.07.003 31582045

[7] Prather K O'Neal Christen M Westrup Alison M Tullos Hurtis J Hughes Kendall L Conner Andrew K. A systematic review of amino acid PET in assessing treatment response to temozolomide in brain glioma. Neuro-oncol Adv. (2022) 4(1):1–14. 10.1093/noajnl/vdac008 PMC8923003 35300149

[8] Gilbert MR Dignam JJ Armstrong TS Wefel JS Blumenthal DT Vogelbaum MA A randomized trial of bevacizumab for newly diagnosed glioblastoma. New Engl J Med. (2014) 370:699–708. 10.1056/NEJMoa1308573 24552317 PMC4201043

[9] Hygino da Cruz LC R.C. Domingues RC Jr Gasparetto EL Sorensen AG. Pseudoprogression and pseudoresponse: imaging challenges in the assessment of posttreatment glioma. AJNR Am J Neuroradiol. (2011) 32:1978–85. 10.3174/ajnr.A2397 21393407 PMC7964401

